# Associations Between Symptoms, Donor Characteristics and IgG Antibody Response in 2082 COVID-19 Convalescent Plasma Donors

**DOI:** 10.3389/fimmu.2022.821721

**Published:** 2022-02-28

**Authors:** Marieke Vinkenoog, Maurice Steenhuis, Anja ten Brinke, J. G. Coen van Hasselt, Mart P. Janssen, Matthijs van Leeuwen, Francis H. Swaneveld, Hans Vrielink, Leo van de Watering, Franke Quee, Katja van den Hurk, Theo Rispens, Boris Hogema, C. Ellen van der Schoot

**Affiliations:** ^1^ Department of Donor Medicine Research, Sanquin Research, Amsterdam, Netherlands; ^2^ Leiden Institute of Advanced Computer Science, Leiden University, Leiden, Netherlands; ^3^ Department of Immunopathology, Sanquin Research, Amsterdam, Netherlands; ^4^ Landsteiner Laboratory, Amsterdam University Medical Centre, University of Amsterdam, Amsterdam, Netherlands; ^5^ Division of Systems Biomedicine and Pharmacology, Leiden Academic Centre for Drug Research, Leiden University, Leiden, Netherlands; ^6^ Department of Transfusion Medicine, Sanquin Blood Supply, Amsterdam, Netherlands; ^7^ Department of Virology, Sanquin Diagnostic Services, Amsterdam, Netherlands; ^8^ Department of Experimental Immunohematology, Sanquin Research and Landsteiner Laboratory Amsterdam University Medical Centre, Amsterdam, Netherlands

**Keywords:** longitudinal, symptoms, antibodies, COVID-19, CCP

## Abstract

Many studies already reported on the association between patient characteristics on the severity of COVID-19 disease outcome, but the relation with SARS-CoV-2 antibody levels is less clear. To investigate this in more detail, we performed a retrospective observational study in which we used the IgG antibody response from 11,118 longitudinal antibody measurements of 2,082 unique COVID convalescent plasma donors. COVID-19 symptoms and donor characteristics were obtained by a questionnaire. Antibody responses were modelled using a linear mixed-effects model. Our study confirms that the SARS-CoV-2 antibody response is associated with patient characteristics like body mass index and age. Antibody decay was faster in male than in female donors (average half-life of 62 versus 72 days). Most interestingly, we also found that three symptoms (headache, anosmia, nasal cold) were associated with lower peak IgG, while six other symptoms (dry cough, fatigue, diarrhoea, fever, dyspnoea, muscle weakness) were associated with higher IgG concentrations.

## Introduction

Severe acute respiratory syndrome corona virus 2 (SARS-CoV-2) emerged late 2019 in China, and by March 2020 was declared a pandemic by the World Health Organization (WHO). As of September 2021, over 200 million individuals have been infected with COVID-19, which has inflicted an immense impact on the healthcare system worldwide. The virus mainly targets the respiratory tract, which can lead from mild symptoms to severe respiratory distress syndrome. Studies have shown that antibody responses against the SARS-CoV-2 spike protein can be first detected 1-3 weeks post symptom onset (1, 2) in most COVID-19 patients and remain in circulation for up to 1 year (3–6). There is however a substantial variation in antibody levels between individuals (5).

Many studies have reported on the association between disease severity and donor characteristics, such as sex, body mass index (BMI), age, and blood group. Males tend to be more susceptible to develop a severe course of the SARS-CoV-2 virus infection (7, 8). In addition, age above 50 and obesity are also associated with increased risk of severe outcome (8–11). ABO blood type may also play a role in COVID-19 infection, but the exact influence remains unclear (12, 13).

Antibody responses also seem to be associated with symptoms and clinical information. In general, SARS-CoV-2 antibody levels are higher in patients with a severe disease outcome (14). A recent study in which COVID-19 convalescent plasma (CCP) donors were followed for three months after symptom resolution showed that greater disease severity, older age, male sex, and high BMI correlate with high SARS-CoV-2 antibody levels (7, 15). The same study also reported that particularly the symptoms fever, body aches, and low appetite correlate with high SARS-CoV-2 antibody levels. Limitations of this study include a small number of subjects and the low number of longitudinal data points available for each subject, which restricts the possibilities to analyse trends in antibody levels over time and the association with donor characteristics and symptoms.

Here, we aimed to gain a more detailed insight into individual symptoms and donor characteristics and their association with the IgG antibody response. Therefore, we analysed a longitudinal data set of 11,118 anti-RBD antibody measurements of 2,082 unique CCP donors. Interestingly, we found that three symptoms (headache, anosmia, nasal cold) were associated with lower peak IgG, while six other symptoms (dry cough, fatigue, diarrhoea, fever, dyspnoea, muscle weakness) were associated with higher IgG concentrations.

## Materials and Methods

### Study Population Samples

Between April 2020 and March 2021, Sanquin Blood Bank (Amsterdam, the Netherlands) collected samples from over 24,000 COVID-19 recovered adults who enrolled in the CCP programme. Within this programme plasma is derived from patients that recovered from COVID-19, with the aim to help patients recover from COVID-19. Donation was voluntary and non-remunerated, and donors provided written informed consent before their first donation. Donors were included based on either a positive PCR or presence of anti-RBD IgG antibodies above 80 Arbitrary Units per ml (AU/ml) and after being free of symptoms for at least two weeks. Donors donated plasma on average every two weeks, until antibody levels were below 4 AU/ml in two consecutive donations. Only donors with at least three consecutive antibody measurements and a complete questionnaire were included in the analyses, resulting in a study population of 2,082 donors (**Supplementary Figure 1**).

### Questionnaire

Starting August 2020, donors that enrolled in the convalescent plasma programme were invited by e-mail to fill out an online questionnaire, programmed in Qualtrics (SAP, Walldorf, Germany). The questionnaire included questions about the possible origin of the infection, the reason why donors were tested and a list of 18 symptoms considered to be COVID-19-related according guidelines specified by the Dutch National Institute for Public Health and the Environment (16). Participants could indicate if they experienced symptoms and, if the symptoms were present, how severe these symptoms were on a 4-point scale, from 1 (very mild) to 4 (severe). Additionally, participants were asked about the duration of their symptoms, whether they consulted a physician or were admitted to hospital and/or intensive care units. The full questionnaire is included as an online supplement. Donors were excluded from analysis if sex, age and/or date of illness was absent.

### Antibody Measurements

IgG to RBD was measured essentially as described before (5, 6). In brief, plates were coated with recombinant RBD, incubated with samples, and bound IgG antibodies were detected using an anti-human IgG antibody (MH16, Sanquin); quantification was done relative to a plasma pool consisting of CCP donors and expressed as AU/mL.

### Statistical Model

Longitudinal trends in antibody levels were analysed with a linear mixed-effects model, using log-transformed anti-RBD IgG levels as outcome variable. Timepoint 0 corresponds to 20 days post onset of symptoms (1). As such, the estimated intercept of the model corresponds to a donor’s estimated peak IgG level (17). The estimated slope of the model is used to calculate a donor’s IgG half-life, in days:


$$t_{1/2}=\log\left(\frac{1}{2}\right)/slope$$

Only measurements within six months post onset of symptoms (1) were included, as in later stages of recovery antibody decline is expected to slow down and no longer expected to follow a loglinear decline (5).

A three-step approach was used to analyse the effects of the covariates. In the first step, a null-model was fit to the data, using time as the only predictor variable and allowing a random intercept and slope to be estimated for each donor. In the second step, we tried to explain the variance in random intercepts and slopes by including fixed effects for donor characteristics, i.e. sex, age, height, weight, BMI, and blood group (ABO and RhD), in addition to the random intercept and slope per donor. In the third step, fixed effects that were statistically insignificant in the second step were removed and additional donor information variables obtained from the questionnaires were added as fixed effects (**Table S1, S2**). This information concerned data on hospitalization, ICU admission, co-morbidities, and the presence of 18 symptoms as shown in **Table 1**. This approach allowed separate estimation of the proportion of variance explained by donor characteristics and clinical information.

**Table 1:** Study population characteristics. Continuous variables are represented by their median and interquartile range (IQR), categorical variables by absolute count and percentage.

Predictor variable	Female		Male	
	Median value or count	IQR or percentage	Median value or count	IQR or percentage
Number of donors (proportion of total)	1236	59.4%	846	40.6%
Number of donations per donor	6	4 – 8	6	4 – 10
Days POS at first donation	48	33 – 77	47	32 – 77
Days POS at last donation*	122	97 - 151	126	103 - 157
Age (years)	45.9	28.0 – 55.3	51.8	39.6 – 59.3
Height (cm)	171	167 – 176	184	180 – 189
Weight (kg)	73	65 – 83	88	80 – 97
BMI (kg/m^2^)	24.8	22.6 – 28.4	26.4	24.0 – 28.2
Blood group ABO				
- A	581	47%	381	45%
- B	120	9.7%	84	9.9%
- O	484	39%	352	42%
- AB	51	4.1%	29	3.4%
Blood group RhD				
- Positive	1024	83%	691	82%
- Negative	212	17%	155	18%
Hospital admission	19	1.5%	50	5.9%
Intensive care	4	0.3%	8	0.9%
Symptoms				
*Asymptomatic*	*8*	*0.6%*	*7*	*0.8%*
Fatigue	979	79%	597	71%
Anosmia/ageusia	853	69%	471	56%
Headache	820	66%	467	55%
Myalgia	705	57%	445	53%
Nasal cold	692	56%	424	50%
Fever	621	50%	507	60%
Dry cough	560	45%	396	47%
Sore throat	519	42%	307	36%
Chills	499	40%	356	42%
Sneezing	461	37%	381	45%
Dyspnoea	461	37%	297	35%
Muscle weakness	426	34%	260	31%
Diarrhoea	221	18%	102	12%
Nausea	184	15%	72	8.5%
Sputum production	178	14%	152	18%
Altered mental status	127	10%	80	9.5%
Skin rash	69	5.6%	27	3.2%
Vomiting	49	4.0%	28	3.3%

*The maximum value is 182, as only donations within six months POS are included.

Significance levels of individual variables were estimated using Satterthwaite’s approximation (18), as degrees of freedom cannot be calculated exactly in models that include both random and fixed effects. Because this approximation is slightly anti-conservative, an alpha-level of 0.01 was chosen to determine statistical significance. Non-significant predictors were excluded after each step. Relative quality, taking into account both goodness of fit and model complexity, of the models was assessed by comparing the Akaike information criteria (AIC) after each step.

Data were processed and analysed with the *R* programming language and environment for statistical computing (version 4.0.3), using packages *lme4* and *lmerTest* for analyses and *ggplot2* for generating graphs.

## Results

### Study Population Characteristics

We used 11,118 antibody measurements of 2,082 unique donors to study the associations between symptoms, donor characteristics, and IgG antibody response. The number of available antibody measurements per donor ranged from 3 to 18 measurements. In addition, each donor completed a questionnaire, which gave insight into symptoms and donor characteristics. **Table 1** shows the distributions of donor and COVID-19 related disease characteristics in the study population.

Compared to all active whole-blood and plasma donors in 2020, donors in our study population are slightly older (46 vs 42 years for women, 52 vs 48 years for men). Median weight and height, as well as proportion of female donors and rhesus D blood group are similar to those of the active donor population. Blood group A is overrepresented in our study population (47% vs 39% for women, 45% vs 39% for men), while blood group O is underrepresented (39% vs 47% for women, 42% vs 47% for men).

### Null-Model Fit (Step 1)

In the first step we estimated an intercept and slope for each individual donor using the null model, describing the linear relationship between log-transformed IgG levels and time post onset of symptoms (1). The residuals, i.e. the difference between measured IgG and predicted IgG as estimated by the null model, follow a normal distribution with mean 0 and standard deviation of 0.21 log (AU/ml). This distribution is independent of time post onset symptoms, supporting the assumption that the relationship is linear after log-transformation. Given this assumption, the estimated peak IgG level set at 20 days POS is most likely an accurate extrapolation and allows for comparisons between donors. **Supplementary Figure 2A** shows the fitted line and actual measurements for four randomly selected donors (donors A to D). **Supplementary Figure 2B** shows the distribution of the residuals over all observations for all donors.

**Figure 1:**
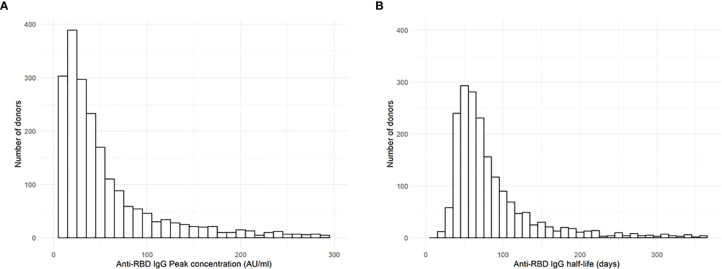
Anti-RBD IgG peak and half-life. **(A)** Distribution of estimated peak IgG concentration (at 20 days POS) and **(B)** estimated half-life of 2,082 COVID convalescent plasma donors, as estimated by the null model. Please note that since both distributions have an extremely long right tail, the horizontal axes are truncated at **(A)** 300 AU/ml and **(B)** 365 days, excluding 70 and 139 donors from left and right histograms, respectively.

After analysing all samples, we found a median peak IgG concentration of 38.8 AU/ml (IQR 20.9-78.6) and a median half-life of 66 days (IQR 50-94) (**Figure 1**). For the majority of donors, the estimated slope corresponds to a plausible antibody half-life. However, for 80 donors (3.8%), the fitted slope was positive, which results in a negative estimated half-life estimate. For an additional 59 donors (2.8%), the estimated half-life is extremely long (defined here as more than 365 days, but estimates ranged up to 16,000 days). This occurs when the estimated slope is very close to zero (but still negative), which may happen when IgG levels barely decrease between measurements and no decay in antibody levels are measured. Examples of donors with a negative half-life and very long half-life are given in **Supplementary Figure 3**. These donors were not excluded from the study in order not to overstate accuracy, and because there was no reason to assume the IgG measurements were incorrect.

### Associations With Predictor Variables (Step 2)

**Figure 2:**
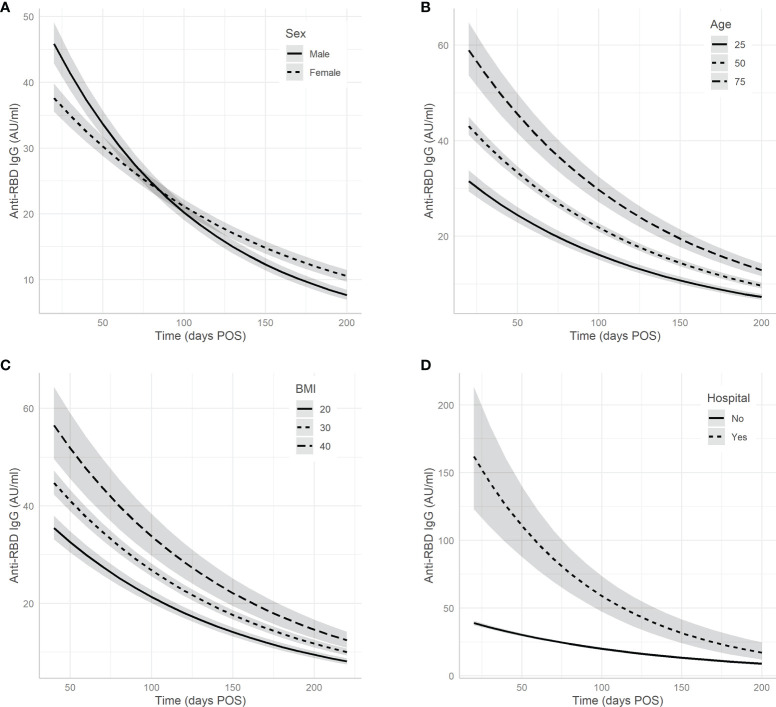
Associations between donor/clinical characteristics and antibody levels. The effects of variables **(A)** sex, **(B)** age, **(C)** BMI, and **(D)** hospital admission on predicted antibody decline. Note that age and BMI are included in the model as continuous predictors; for clarity, the associations are only plotted for three values. Grey bands represent 95% confidence intervals.

The results of step 2, where individual donor characteristics were added to the model as predictor variables, are shown in **Figures 2A–C** and **Table 2**. Sex was associated with the slope (**Figure 2A**), as the rate of antibody decay is faster in men: the median slope for men corresponds to a half-life of 62 days, while this is 72 days for women. Men displayed higher peak IgG levels than women, but this difference was not statistically significant (p = 0.68). Age (**Figure 2B**) and BMI (**Figure 2C**) were both positively correlated with peak IgG concentration. A one-year increase in age corresponds to a 0.013 increase in the log-transformed IgG level, an increase of one BMI point corresponds to a 0.024 increase in log-transformed IgG level. No significant associations with antibody titres were found for variables blood group, height, and weight. Random effects for peak IgG level and antibody half-life are positively correlated with a correlation coefficient of 0.29, indicating that higher peak IgG is moderately associated with higher (less negative) slope, and therefore with a longer half-life.

**Table 2:** Point estimates and 95% confidence intervals of fixed effects on log-transformed IgG levels.

Estimates of average intercept (log peak IgG) and slope		
Term	Estimate	95% CI
Intercept (log peak IgG)	2.382	2.274 – 2.490
Slope [Δlog(IgG) per day]	-0.010	-0.011 – -0.010
**Fixed effects on the intercept**	**Estimate**	**95% CI**
*Sex: female	*-0.017*	*-0.063 – 0.096*
Age (per 10 years increase)	0.128	0.100 – 0.157
BMI (per 5 points increase)	0.119	0.075 – 0.164
Hospital admission: yes	1.156	0.934 – 1.379
Headache: yes	-0.113	-0.193 – -0.032
Anosmia: yes	-0.111	-0.189 – -0.033
Nasal cold: yes	-0.101	-0.177 – -0.025
Dry cough: yes	0.095	0.019 – 0.171
Fatigue: yes	0.140	0.044 – 0.236
Diarrhoea: yes	0.148	0.043 – 0.252
Muscle weakness: yes	0.172	0.083 – 0.261
Shortness of breath: yes	0.196	0.111 – 0.280
Fever: yes	0.228	0.149 – 0.308
**Fixed effects on the slope**	**Estimate**	**95% CI**
Sex: female	0.003	0.002 – 0.004
Hospital admission: yes	-0.004	-0.007 – -0.001

*The effect of sex on the intercept (peak IgG) was not statistically significant, but the variable is not excluded from the model due to its effect on the slope.

### Associations with Clinical Information (Step 3)

**Figure 3:**
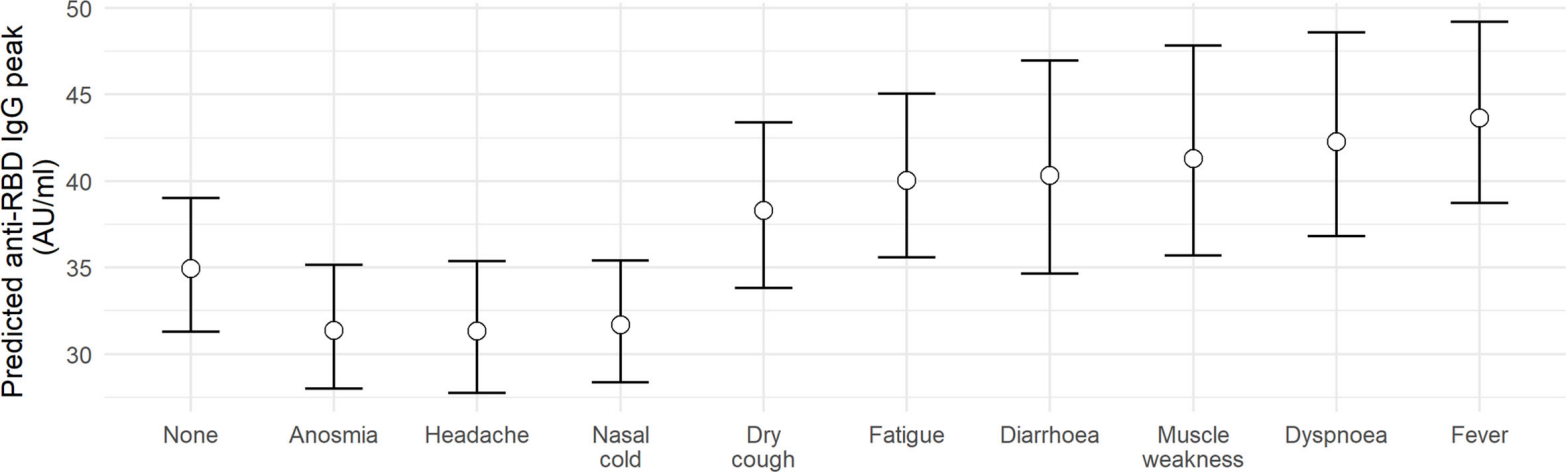
Predicted impact of various symptoms on anti-RBD IgG peak concentration. Estimated peak IgG concentrations when different symptoms are displayed. For each of the symptoms here, the difference in peak IgG as compared to the group without this symptom is statistically significant with p < 0.001.

**Figure 4:**
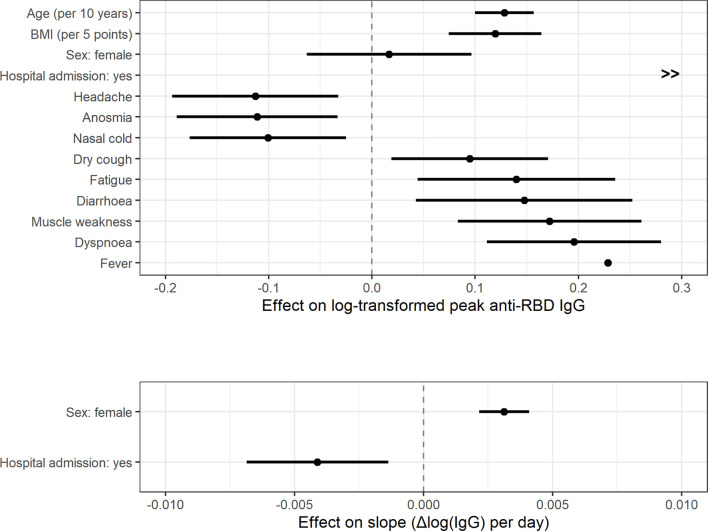
Effect size and 95% confidence intervals of fixed effects on anti-RBD IgG peak concentration (log-transformed) and the slope.

After adding clinical information significant associations with peak IgG concentration were found for hospital admission and various clinical symptoms (**Figures 2D**, **3**, **4** and **Table 2**). Hospital admission was significantly associated with both higher peak IgG level and shorter half-life (**Figure 2D**). Nasal cold, headache, and anosmia were associated with lower peak IgG levels, while dry cough, fatigue, fever, dyspnoea, diarrhoea, and muscle weakness were associated with higher peak IgG levels. **Figure 3** shows the estimated peak IgG level when these symptoms are present. Note that values on the y-axis are the predicted peak IgG levels when all continuous variables are equal to their average value, and all binary variables (hospital admission and all other symptoms) equal zero.

The largest difference was found for the ‘hospital admission’ variable. Donors admitted to the hospital had considerably higher antibody levels, with an estimated difference of 77.8 AU/ml on the peak IgG concentration. These donors also have a faster rate of antibody decay, corresponding to an estimated half-life of 48 days (95% CI: 40-58 days) for men, and 60 days (95% CI: 49-80 days) for women.

### Variance Explained by Model

In the null-model that was fitted in step 1 (without any fixed effects), all variation in peak IgG and half-life was attributed to the individual variation per donor. As fixed effects were added in step 2, part of this variation was now explained by these fixed effects, and the variation explained by the random effects decreased. **Table 3** shows the variance of the random effects per donor in the null-model, as well the variance of the random effects as after adding donor characteristics as covariates (step 2), and after adding the clinical information (step 3). The variance reduction relative to the null-model (step 1) by the addition of extra explanatory variables in each step is also provided. Model fit was compared using the Akaike Information Criterion (AIC) and tested for statistical significance using a nested ANOVA, results of which are shown in **Table 3**.

**Table 3:** Variance of random effects in models of all three steps. Percentual variance decrease relative to the null-model is given in brackets.

Model	Variance of random effect on peak log(IgG)(reduction relative to the null-model)	Variance of random effect on slope Δlog(IgG)/day(reduction relative to the null-model)	AIC (ANOVA p-value relative to previous step)
Step 1: null-model	0.8814	0.0497	11886
Step 2: donor characteristics	0.7758 (-12%)	0.0485 (-2.4%)	11615 (p < 0.001)
Step 3: donor characteristics + clinical information	0.6610 (-25%)	0.0481 (-3.2%)	11290 (p < 0.001)

## Discussion

In this retrospective observational study, we investigated potential associations between SARS-CoV-2 specific antibody kinetics and various donor characteristics and COVID-19 symptoms. To our knowledge, this is currently the largest study that describes such associations. Individual antibody responses were modelled using a linear mixed-effects model, from which peak IgG concentration and antibody half-life were determined. Symptoms and donor characteristics were obtained from a questionnaire. Our study shows that the SARS-CoV-2 antibody response is associated with patient characteristics like sex, age, and BMI. Of note, we also found that specific COVID-19 symptoms are associated with antibody levels.

As reported earlier, we found a large variation in anti-RBD antibody peak levels. A strength of our study are the longitudinal measurements, which enabled us to reliably estimate the peak level of each individual donor independent on the timing of the first antibody measurement. Only a quarter of the variation in peak IgG concentration between patients can be explained by associations with donor characteristics and disease symptoms. To a lesser degree, donor characteristics were also associated with differences in antibody half-life, which was also variable between donors, albeit less than the peak level. The antibody half-life was not correlated to peak levels. Whether these differences in antibody half-life reflect differences in protection for reinfection will be investigated, and this thoroughly characterized donor cohort can serve as bench mark for those studies.

Six symptoms (dry cough, fatigue, diarrhoea, fever, dyspnoea, muscle weakness) were associated with higher IgG concentrations and three symptoms (headache, anosmia, nasal cold) were associated with lower peak IgG concentrations against RBD. This association between symptoms and antibody levels may possibly reflect the fact that the SARS-CoV-2 virus frequently initiates infection in the upper airways (mild symptoms and low IgG levels) before spreading through the body (severe symptoms and high IgG levels). Headache, anosmia and nasal cold were common symptoms, each present in at least 50% of patients in our population. Fatigue was present in more than 70% of patients and was associated with higher peak IgG concentration, suggesting more severe illness. A previous study in a hospital cohort found that fatigue and dyspnoea are prognostic for severe infection, and a stuffed nose (comparable to nasal cold) for mild infection, which is in line with our findings (19).

Furthermore, we found higher age and BMI to be associated with higher peak IgG concentrations. Sex was not associated with peak IgG concentration, but men had significantly shorter antibody half-lives than women (62 vs 72 days respectively). The small group of patients that had been admitted to hospital displayed both higher peak IgG concentrations and shorter half-lives. Probably this effect is the result of the presence of short-lived plasmablasts that produce high levels of antibodies. Previous studies found sex differences in COVID-19 immune responses, with higher IgG concentrations associated with male sex, older age, and hospitalization (20–22). Although our results are consistent with these findings for age and hospitalization, we found that the association between male sex and higher peak IgG concentration was not significant after correction for age and BMI. This suggests that the previously found association with male gender was possibly due to the increased risk of severe disease in men. Most studies on differences in antibody response are performed in hospital cohorts, our study population consisted mainly of recovered patients that were not admitted to hospital (96.7%), and therefore disease severity is expected to be lower. Consistently, BMI in the non-hospitalized group was 25.9 compared to 28.8 in hospitalized patients.

A strength of our study is the large number of recovered patients included in our study population. The status of Sanquin as the only blood bank in the Netherlands, combined with well-established connections with municipal health services, allowed us to invite people with a positive PCR test to become CCP donors after recovery. This allowed us to both include non-hospitalized and hospitalized patients in the cohort. However, we could only include donors who were healthy enough to regularly donate plasma, which means that our results are mainly applicable towards patients with a mild outcome. As a result our study is more representative of the total COVID-19 patient population than studies on hospitalized patient cohorts. It should also be noted that some bias may be present in our data, as symptoms are self-reported by patients after recovery. Relatively mild symptoms, such as nasal cold, may therefore be underreported by patients who at the same time experienced more severe symptoms, such as fever or dyspnoea. However, this explanation is unlikely to negate the association we found, as all symptoms associated with lower peak IgG were present in more than 50% of patients.

In conclusion, our study indicates that several COVID-19 symptoms are associated with SARS-CoV-2 antibody levels in addition to the previously described association with sex, age, and BMI. Discovery of these associations aids us in understanding why antibody responses differ between patients. The predictive value of IgG concentrations could also be used by blood banks to pre-select individuals with high and/or stable antibody levels as potential CCP donors.

## Data Availability Statement

Restrictions apply to the datasets: The datasets presented in this article are not readily available because they contain personal information. Requests to access the datasets should be directed to the corresponding author.

## Ethics Statement

The studies involving human participants were reviewed and approved by Ethische Adviesraad van Stichting Sanquin Bloedvoorziening, Stichting Sanquin Bloedvoorziening, Plesmanlaan 125, 1066 CX, Amsterdam, The Netherlands. The patients/participants provided their written informed consent to participate in this study.

## Author Contributions 

CES, MJ, and TR conceived the study. AB, JC, MJ, ML, MV, FS, HV, LW, FQ, KH, TR, BH, and CES were involved in study design and organization. MV conducted experiments. MV and MJ analyzed data. MV and MS wrote the paper. MJ, TR, and CES supervised the study. All authors provided critical revision of the paper. All authors contributed to the article and approved the submitted version.

## Funding

This study was supported by the European Commission (SUPPORT-E, grant number 101015756), by ZonMW (Protective Immunity, grant number 10430 01 201 0012), and Sanquin Blood Supply Foundation, PPOC grant 18-14/L2337.

## Conflict of Interest

The authors declare that the research was conducted in the absence of any commercial or financial relationships that could be construed as a potential conflict of interest.

## Publisher’s Note

All claims expressed in this article are solely those of the authors and do not necessarily represent those of their affiliated organizations, or those of the publisher, the editors and the reviewers. Any product that may be evaluated in this article, or claim that may be made by its manufacturer, is not guaranteed or endorsed by the publisher.
