# Supplementary material for: Associations Between Symptoms, Donor Characteristics and IgG Antibody Response in 2082 COVID-19 Convalescent Plasma Donors
Source: Front Immunol. 2022 Feb 28;13:821721. doi: 10.3389/fimmu.2022.821721 (PMC8918483; doi:10.3389/fimmu.2022.821721)
Supplement: Supplementary file 1 [file DataSheet_1.pdf]

## Supporting information

# Associations between symptoms, donor characteristics and IgG antibody response in 2082 COVID-19 convalescent plasma donors

Running title: Associations between symptoms, donor characteristics and SARS-CoV-2 IgG antibody levels

Marieke Vinkenoog[1,4], Maurice Steenhuis[2], Anja ten Brinke[2], J.G. Coen van Hasselt[3], Mart P. Janssen[1,4], Matthijs van Leeuwen[4], Francis Swaneveld[5], Hans Vrielink[5], Leo van de Watering[5], Franke Quee[1], Katja van den Hurk[1], Theo Rispens[2], Boris Hogema[6], C. Ellen van der Schoot[7]

### Affiliations

[1]Department of Donor Medicine Research, Sanquin Research, Amsterdam, The Netherlands.
[2]Department of Immunopathology, Sanquin Research, Amsterdam, The Netherlands, and Landsteiner Laboratory, Amsterdam University Medical Centre, University of Amsterdam, Amsterdam, The Netherlands.
[3]Division of Systems Biomedicine and Pharmacology, Leiden Academic Centre for Drug Research, Leiden University, Einsteinweg 55, 2333 CC, Leiden, The Netherlands.
[4]Leiden Institute of Advanced Computer Science, Leiden University, Leiden, The Netherlands.
[5]Department of Transfusion Medicine, Sanquin Blood Supply, 1066 CX Amsterdam, the Netherlands.
[6]Department of Virology, Sanquin Diagnostic Services, Amsterdam, The Netherlands.
[7]Department of Experimental Immunohematology, Sanquin Research and Landsteiner Laboratory Amsterdam University Medical Centre, 1066 CX Amsterdam, the Netherlands.

### Corresponding author

C. Ellen van der Schoot; Department of Experimental Immunohematology, Sanquin Research and Landsteiner Laboratory Amsterdam University Medical Centre, 1066 CX Amsterdam, the Netherlands; e.vanderschoot@sanquin.nl.

### Keywords

Longitudinal; symptoms; antibodies; COVID-19; characteristics; CCP

**Questionnaire anti-SARS-CoV-2 donors**

Note: the original questionnaire was in Dutch, this is a translated version.

Q1: What is your donor ID? You can find this in the accompanying email.

Q36: What is your date of birth?

Day: ___

Month: ___

Year: ___

Q2: What is your sex?

- o Male
- o Female

Q3: How would you describe your COVID-19 status?

- o I suspect I have had COVID-19 because I have had a positive PCR test.
- o I suspect I have had COVID-19 because antibodies have been detected in my blood.
- o Other, namely: _______

Q4: Where did you contract the infection?

- o In the Netherlands
- o Abroad

Q5 (if Q4 answered with 'abroad'): In which country did you contract the infection?

Q6: Why were you tested for presence of the coronavirus?

- o Because I was ill/had symptoms
- o Because I was in contact with a (possibly) infected person
- o Because of my occupation (health care, contact profession)
- o Different: _______

Q7: Did you experience the following symptoms?

- ☐ Nasal cold/coryza
- ☐ Sore throat
- ☐ Dry cough
- ☐ Fatigue
- ☐ Sputum production
- ☐ Muscle or joint ache
- ☐ Headache
- ☐ Fever
- ☐ Shortness of breath
- ☐ Diarrhoea
- ☐ Nausea
- ☐ Vomiting
- ☐ Chills
- ☐ Sneezing
- ☐ Skin rash
- ☐ Feeling confused
- ☐ Muscle weakness
- ☐ Loss of/less smell or taste

Q8-25 (for each symptom answered with 'yes' in Q7): How much were you affected by this symptom?

- Very mildly affected
- Mildly affected
- Moderately affected
- Severely affected

Q37: Did you have pneumonia?

- Yes
- No

Q26: When did your symptoms start? If you don't remember exactly, please make an estimate.

Day: ___
Month: ___
Year: ___

Q27: When did your symptoms end? If you don't remember exactly, please make an estimate.

Day: ___
Month: ___
Year: ___

Q28: Were you admitted to hospital for these symptoms?

- Yes
- No

Q29 (if Q28 is 'yes'): On which date were you admitted to hospital?

Day: ___
Month: ___
Year: ___

Q30 (if Q28 is 'yes'): Were you admitted to intensive care?

- Yes
- No

Q31 (if Q30 is 'yes'): How many days were you in intensive care in total?

Q32 (if Q28 is 'yes'): Were you given extra oxygen?

- Yes
- No

Q33 (if Q28 is 'yes'): Did you receive artificial ventilation?

- Yes
- No

Q34 (if Q28 is 'yes'): On which date were you discharged from the hospital?

Day: ___
Month: ___
Year: ___

Q35: Are you in one or more of the following risk groups?

- ☐ People aged 70 or older
- ☐ People with chronic airway or lung disease and under treatment by pulmonologist
- ☐ Chronic heart disease patients under treatment by cardiologist
- ☐ People with diabetes that is not well regulated and/or with complications
- ☐ People with kidney disease who need dialysis or are waiting for a kidney transplantation
- ☐ People with lowered immunity to infection due to medication use for auto-immune disease
- ☐ People who have had an organ or stem cell transplantation
- ☐ People without a spleen or without a functioning spleen
- ☐ People with a blood disease
- ☐ People with lowered immunity due to immunity-lowering medication
- ☐ Cancer patients who have had chemotherapy and/or radiation in the past 3 months
- ☐ People with severe immune disorder that requires medical treatment
- ☐ People with HIV infection who are not (yet) under treatment, or with HIV infection with CD4 under 200/$mm^2$
- ☐ People with severe liver disease
- ☐ People with a BMI over 40

Q38: If there is anything you would like to add, or explain an answer further, please do so here:

__________

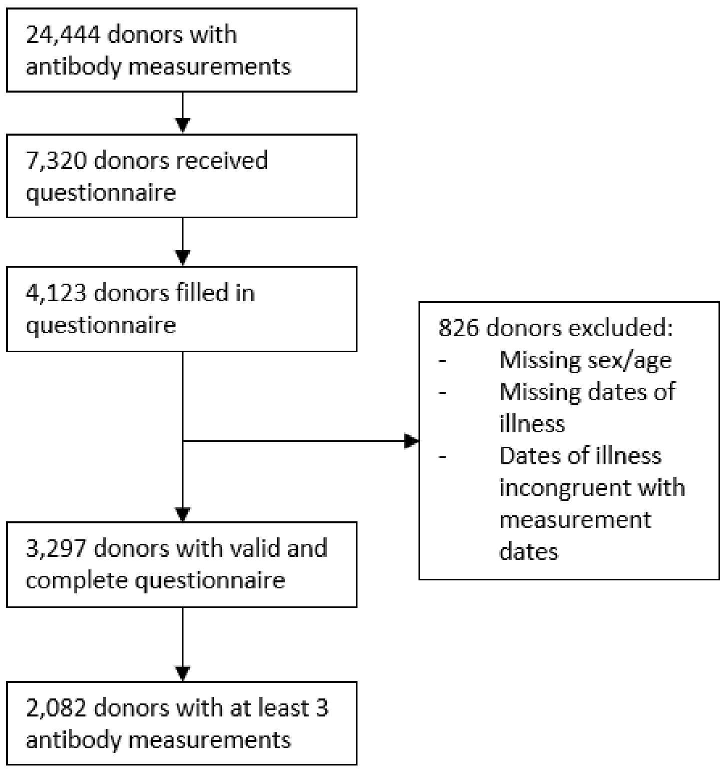


**Supplemental Figure 1. Flowchart showing the criteria for inclusion and exclusion of donors.**

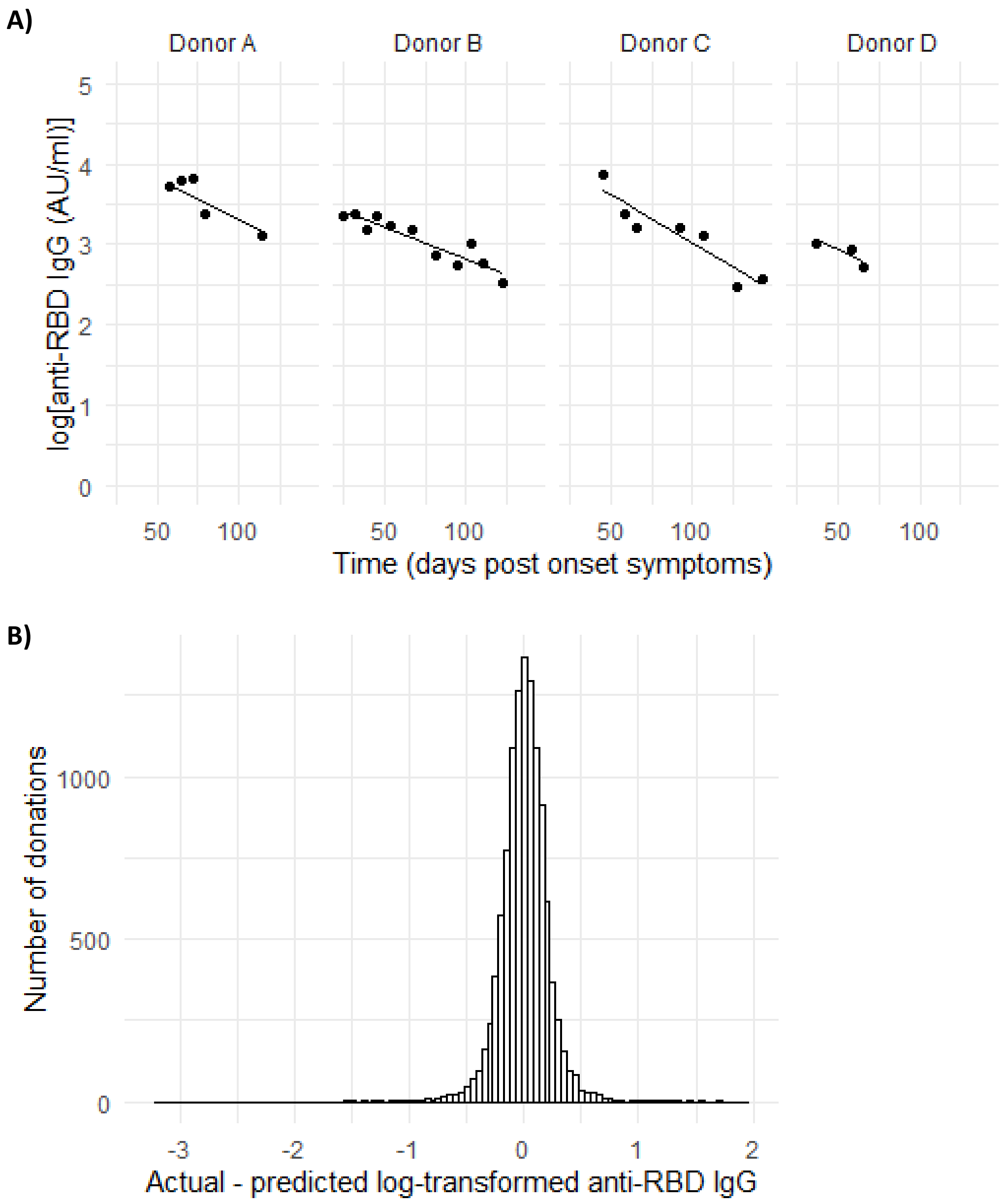


**Supplemental Figure 2. Null model fit (step 1).** (**A**) Measured anti-RBD IgG levels (points) and fitted line as estimated by the linear model for four randomly selected donors, with (**B**) distribution of residuals over all observations, for all donors.

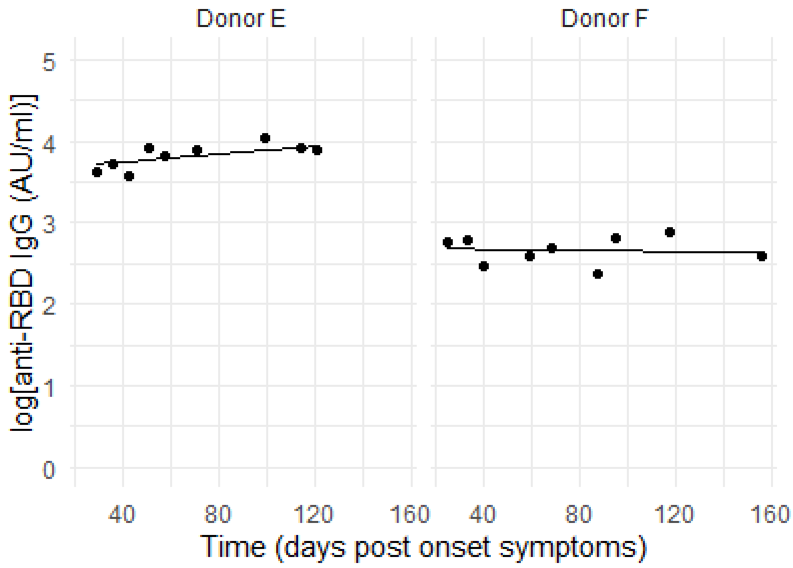


**Supplemental Figure 3. No decay in antibody levels.** Example of a donor with increasing IgG levels (left panel) and one with near-constant IgG levels (right panel). Estimated slopes for these donors are 0.0024 and -0.0151, corresponding to estimated half-lives of -292 and 1843 days, respectively.

**Table S1. Sum of squares and p-values of fixed effects after step 2, calculated by backward stepwise reduction**

| **Fixed effect on intercept** | **Sum of squares** | **P-value** |
|---|---|---|
| Weight | 0.0070 | 0.762 |
| Blood group ABO | 0.4456 | 0.121 |
| Height | 0.3630 | 0.030 |
| Blood group RhD | 0.4638 | 0.014 |
| BMI | 3.888 | <0.001 * |
| Age | 8.752 | <0.001 * |
| **Fixed effect on slope** | **Sum of squares** | **P-value** |
| BMI | 0.002 | 0.890 |
| Age | 0.004 | 0.831 |
| Height | 0.009 | 0.735 |
| Weight | 0.035 | 0.500 |
| Blood group ABO | 0.188 | 0.483 |
| Blood group RhD | 0.085 | 0.294 |
| Sex | 3.403 | <0.001 * |

**Table S2. Sum of squares and p-values of fixed effects after step 3, calculated by backward stepwise reduction**

| **Fixed effect on intercept** | **Sum of squares** | **P-value** |
|---|---|---|
| Sneezing | 0.000 | 0.971 |
| Vomiting | 0.004 | 0.812 |
| Confusion | 0.011 | 0.700 |
| Coughing up mucus | 0.084 | 0.296 |
| Throat ache | 0.120 | 0.210 |
| Joint/muscle ache | 0.251 | 0.070 |
| Nausea | 0.247 | 0.072 |
| Intensive care admission | 0.274 | 0.059 |
| Shivers | 0.345 | 0.034 |
| Skin rash | 0.451 | 0.015 |
| Anosmia | 0.645 | 0.004 * |
| Fatigue | 0.567 | 0.003 * |
| Nasal cold | 0.449 | 0.002 * |
| Dry cough | 0.394 | 0.002 * |
| Diarrhoea | 0.462 | 0.001 * |
| BMI | 2.000 | <0.001 * |
| Hospital admission | 8.555 | <0.001 * |
| Fever | 1.836 | <0.001 * |
| Shortness of breath | 1.510 | <0.001 * |
| **Fixed effect on slope** | **Sum of squares** | **P-value** |
| Sex | 3.470 | <0.001 * |
